# Winter Frosts Reduce Flower Bud Survival in High-Mountain Plants

**DOI:** 10.3390/plants10081507

**Published:** 2021-07-22

**Authors:** Johanna Wagner, Karla Gruber, Ursula Ladinig, Othmar Buchner, Gilbert Neuner

**Affiliations:** Department of Botany, Functional Plant Biology, University of Innsbruck, Sternwartestrasse 15, A-6020 Innsbruck, Austria; karlagrbr@gmail.com (K.G.); ursula.ladi@gmail.com (U.L.); mail@o.buchner.co.uk (O.B.)

**Keywords:** alpine plants, climate change, ice nucleation, ice propagation pattern, freezing stress, frost resistance, freezing tolerance, winter buds, winter snow cover

## Abstract

At higher elevations in the European Alps, plants may experience winter temperatures of −30 °C and lower at snow-free sites. Vegetative organs are usually sufficiently frost hardy to survive such low temperatures, but it is largely unknown if this also applies to generative structures. We investigated winter frost effects on flower buds in the cushion plants *Saxifraga bryoides* L. (subnival-nival) and *Saxifraga moschata* Wulfen (alpine-nival) growing at differently exposed sites, and the chionophilous cryptophyte *Ranunculus glacialis* L. (subnival-nival). Potted plants were subjected to short-time (ST) and long-time (LT) freezing between −10 and −30 °C in temperature-controlled freezers. Frost damage, ice nucleation and flowering frequency in summer were determined. Flower bud viability and flowering frequency decreased significantly with decreasing temperature and exposure time in both saxifrages. Already, −10 °C LT-freezing caused the first injuries. Below −20 °C, the mean losses were 47% (ST) and 75% (LT) in *S. bryoides*, and 19% (ST) and 38% (LT) in *S. moschata*. Winter buds of both saxifrages did not supercool, suggesting that damages were caused by freeze dehydration. *R. glacialis* remained largely undamaged down to −30 °C in the ST experiment, but did not survive permanent freezing below −20 °C. Winter snow cover is essential for the survival of flower buds and indirectly for reproductive fitness. This problem gains particular relevance in the context of winter periods with low precipitation and winter warming events leading to the melting of the protective snowpack.

## 1. Introduction

High mountains are stress-dominated habitats. Various abiotic stress factors such as temperatures extremes, strong irradiation, drought, long snow duration, short growing seasons, strong winds, shallow soils and mechanical strains operate as selection filters and adaption forces on plants [1]. In detail, stress conditions strongly differ depending on the climate zone, the elevation and the (micro-)topography. For instance, drought limits plant growth in arid mountains of central Asia [2], and mountains with a Mediterranean-type climate where summer drought is critical [3,4,5]. Heat may become effective in wind-sheltered microhabitats when, due to strong solar irradiation, the short vegetation heats up considerably above air temperature [6,7,8]. Snow cover duration and thus the period available for growth and seed production mainly shapes plant communities in the mountains of temperate and cold climates [9,10,11].

Frost is one of the few common environmental factors limiting plant growth at higher elevations across the globe. Depending on the geographical location and elevation, severe frosts occur regularly (tropical, subtropical and arid mountains) or mainly during winter (temperate-zone mountains). As frost is a selective factor, high-mountain plants are adapted to predictable temperature regimes in their respective environment but are at risk of suffering from frost damage in the case of untimely cold spells and/or insufficient snow protection. In temperate mountains, the situation is particularly critical at the beginning of the growing season when frost dehardened plants are struck by heavy spring frosts as documented, e.g., for some wildflower species in the Rocky Mountains [12], and dwarf-shrubs [13] and several alpine grassland species [1] in the Swiss Alps. However, episodic cold spells with snowfalls in summer may also cause frost damage when nights are clear and the snow cover is too shallow, melts during the day, or is absent at windblown ridges and in steep terrain [1,14,15,16]. In the Austrian Alps, during cold spells in the summer months, June–August, free air temperatures may drop down to −8 °C in the alpine zone and to nearly −15 °C in the nival zone [17]. Near the ground, unprotected plants may experience significantly lower temperatures due to night-time radiative energy loss [16,18,19]. Such low temperatures fall below the frost damage threshold of most species in the respective habitat [1,15,19,20,21]. Generative tissues in particular are at risk of being severely damaged because they are significantly more frost susceptible than vegetative tissues [19,22,23]. The frost damage risk, calculated on the basis of the frequency and severity of unpredictable summer frosts and freezing resistance of generative structures, turned out to be moderate in the alpine zone but high for species growing at the upper boundary of distribution in the nival zone [19].

During winter, air temperatures in the Austrian Alps regularly drop below −20 °C in the alpine zone, and to around −30 °C in the nival zone. In extreme cold winters, temperatures down to −35 °C are possible [17]. Winter frosts do not affect plants below deep snow where they experience constant temperatures of between 0 and −5 °C [24,25,26,27,28]. Without snow cover, however, plants are fully exposed to free air temperatures or below due to radiative cooling [18]. Cold acclimation provides sufficient protection against winter frost damage in most species [29,30,31,32,33]. In herbaceous plants, the cold acclimation is triggered by periodic temperatures close to zero in autumn. Increased incorporation of cold-stable phospholipids into the biomembranes and the accumulation of frost protection proteins and low-molecular substances such as soluble carbohydrates reduce the freeze-induced stress [34]. Fully frost-hardened plants mostly tolerate temperatures far below the naturally occurring air temperature minima; single species survive even the immersion in liquid nitrogen [31,33].

To survive subzero temperatures, cold-adapted plants may either avoid tissue freezing by supercooling or tolerate extracellular ice formation and the associated freeze-dehydration to a certain extent [31,35,36,37,38,39]. In summer, most high-mountain plants tolerate extracellular ice in vegetative aboveground organs as long as freezing temperatures are not too extreme and do not act over a long time period. Generative structures, however, are ice-sensitive, particularly during bolting, anthesis and early fruiting [19,22,40]. There are only rare exceptions, such as *Ranunculus glacialis* L., whose generative structures remain ice-tolerant during all flowering and fruiting stages [19]. Generative shoots of cushion plants freeze independently: upon ice nucleation in a single shoot, mostly at the shoot basis or in the stalk, ice quickly propagates throughout the entire shoot inclusive flowers, but not into neighboring shoots. Frozen flowering shoots suffer full damage. Since not all shoots freeze at the same time and anatomical ice barriers were not detected, a thermal ice barrier in the densely leaved shoot bases was assumed, which keeps single inflorescences supercooled for some time, and thus, undamaged [40].

Frost survival mechanisms of mountain plants in winter have—with the exception of some obligatory chionophytes that require snow protection in winter—been mainly studied in woody plants whose shoots outside a constant snow cover are not only subjected to severe freezing temperatures but also to frequent freeze–thaw cycles and frost desiccation [31,41]. Generative and vegetative buds of most temperate woody plants survive winter frosts by supercooling [31,42]. Supercooling is aided by (1) structural ice barriers that restrict ice propagation from the frozen stem into ice susceptible generative [43,44] and vegetative buds [42,45], and (2) species-specific extents of freeze dehydration. During freeze dehydration, water of supercooled bud tissues migrates to extra-organ ice masses that form either in bud scales or in the stem below [46,47,48]. In 50% of studied temperate species, ice masses even formed between the supercooled young leaves of the bud [42]. In *Alnus alnobetula* (Ehrh.) K. Koch, lipophilic excretions of the young leaves prevent extrinsic ice nucleation and allow buds to be maintained in a deep supercooled state [49].

Our knowledge about winter freezing resistance in high-mountain plants refers nearly exclusively to vegetative organs. It is virtually unknown whether flower buds in winter are similarly frost hardy as vegetative structures, or—as during the growing season—tolerate less frost. Flower bud survival in winter is essential, as it determines the flowering frequency in summer, and thus indirectly, the seed output of a plant [12]. The majority of perennial high-mountain plants initiates flower buds in the year before anthesis by the conversion of vegetative shoot apical meristems into floral meristems, which develop further into single flowers or inflorescences [50,51,52,53,54]. Some species initiate flowers even two or more years before flowering [50,55,56]. Depending on the species, flower buds pass winter in different developmental states from early primordial to highly differentiated. The more developed the flower buds are, the earlier they start flowering and the greater the chance to mature seeds during the short alpine summer [52]. Buds of herbaceous plants usually overwinter just below the soil surface, where they are protected from direct frost [1]. The flower buds on the twigs of dwarf shrubs and in cushion plants are less protected. In the latter, generative buds are in a terminal or lateral position on aboveground short-stem shoots enclosed by inwardly bent foliage leaves.

The present study investigates winter freezing resistance of generative buds and compares it to that of vegetative plant structures in three common high-mountain species from the European Central Alps growing at sites with different site preferences. *Saxifraga moschata* Wulfen is a perennial semi-evergreen cushion plant occurring from the alpine to the nival zone at sites with more or less snow protection in winter, the subnival–nival cushion plant *Saxifraga bryoides* L. mostly grows at exposed sites with no or little snow cover, and *Ranunculus glacialis* L., a subnival–nival cryptophyte, prefers snow-rich sites. The study species also differ in their winter status of flower bud development, which is mainly primordial to middle (all organs basically formed) in *S. bryoides*, primordial to advanced in *S. moschata*, and advanced in *R. glacialis*. We tested to what extent the duration and intensity of freezing temperatures in winter affect bud survival and flowering frequency in summer.

Specifically, we addressed the following questions: (1) is there a relationship between growing site preference and winter freezing resistance? (2) Are there differences in winter freezing resistance between generative and vegetative structures? (3) Are there differences in the frost susceptibility with regard to the developmental status of the flower buds? (4) At what temperatures does ice nucleation and the subsequent ice propagation in cushion plants start, what is the freezing pattern in winter, and is there a relationship between nucleation temperatures and freezing damage? Finally, (5) do winter frosts affect flowering frequency in summer?

We expected that the cushion plants, which do not rely on a permanent snow cover in winter, would tolerate frost better than the chionophilous species *R. glacialis*. Similar to what has been shown for the summer freezing resistance [19], flower buds were anticipated to be at greater risk of suffering frost damage than vegetative parts. Since the tissues of the floral apices in an early primordial developmental state are not yet specifically differentiated, we expected early stages to be less frost susceptible than middle and late developmental stages. Finally, we assumed that winter frost damage in flower buds should result in less flowers per individual in summer.

## 2. Results

A short overview of the experiments performed at this point should help to better understand the results. Potted plants were exposed to temperatures between −10 and −30 °C in temperature-controlled freezing chambers for one night or for one week. In addition, ice nucleation temperatures in the buds of the cushion plants were recorded by IDTA (infrared differential thermal analysis). Buds were examined for frost damage using vital staining. In a regrowth experiment, flowering frequency was determined at the time of anthesis.

### 2.1. Effects of Winter Frost on Saxifraga moschata

**Figure 1:**
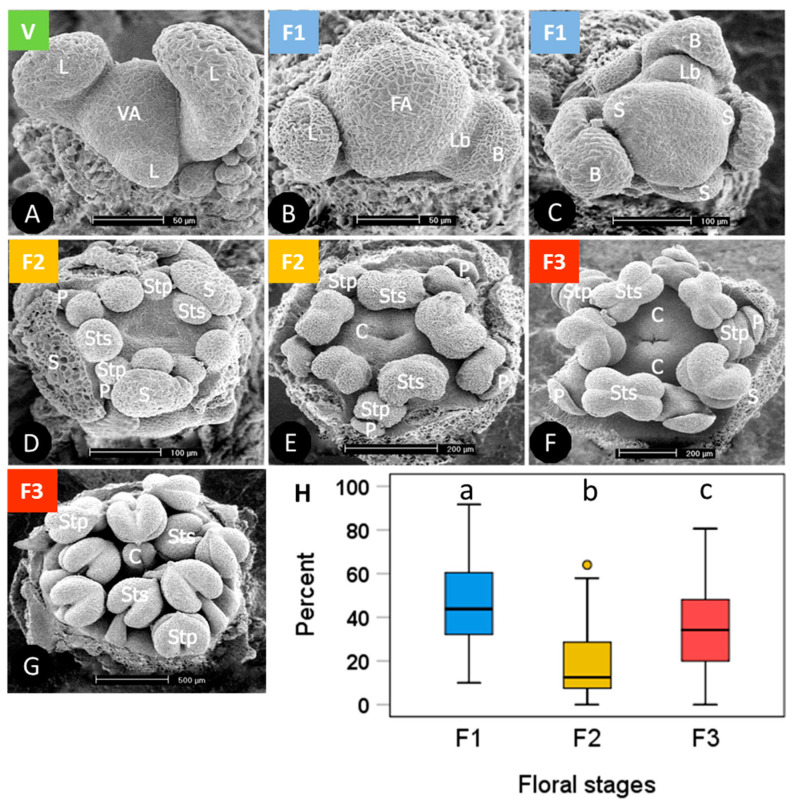
*Saxifraga moschata* Wulfen. Developmental stages of floral buds in winter dormant individuals at the time of the freezing experiments: green: vegetative (V); blue: early floral stages (F1); yellow: floral middle stages (F2); red: floral late stages (F3). (**A**) vegetative apex (VA) with leaf primordia (L) in alternate position; (**B**) floral apex (FA) shortly after the transition from vegetative to floral and lateral bud (Lb) with bract (**B**); (**C**) the apical dome has further flattened and sepal primordia (S) have emerged; (**D**) sepal, and petal (P) primordia and two whorls of stamens in episepal (Sts) and epipetal (Stp) position have formed; (**E**) Carpel (C) margins appear as a crater-like structure. Stamen primordia have started to differentiate into filaments and anthers. Sepals that had begun to conceal the flower within had been removed; (**F**) elongating carpels have formed a cone, pollen sacs are clearly distinguishable; (**G**) carpel tips (c) have bent outside to form stigma lobes. Inside the carpels ovule primordia have emerged along the placental ridge (not visible in the picture); sepals and petals were removed. (**H**) percentage range of floral buds in different stages per individual; boxes show the median (line inside box), the 25th and 75th percentile (top and bottom of box), maximum and minimum values within the 1.5 times the interquartile range (whiskers), and outliers (circle); different lowercase letters indicate statistical differences among floral stages (*n* = 53 individuals, F1 > F3 > F2, *p* ≤ 0.007, one-way ANOVA). Photos with permission of I. Larl.

Terminal buds randomly sampled from individual plant cushions in winter were on average 54% ± 9 SD (standard deviation) vegetative and 46% ± 9 SD floral. Flower buds were in different phases from early primordial to all floral organs in an advanced developmental state (Figure 1). For the stage-dependent analysis of bud viability, the development status was assessed as vegetative (Figure 1A), floral early (floral apex flattened and sepal primordia emerged, F1 in Figure 1B,C), floral middle (sepal and petal primordia and two whorls of stamens formed to early carpel development, F2 in Figure 1D,E), and floral late (advanced development of all floral organs, F3 in Figure 1F,G). On average, 46% of the floral buds were in an early, 19% in a middle and 35% in a late floral stage (Figure 1H). Though the ratios varied greatly within individuals, mean proportions differed significantly among developmental stages (*p* ≤ 0.007, one-way ANOVA, Duncan post hoc test).

**Figure 2:**
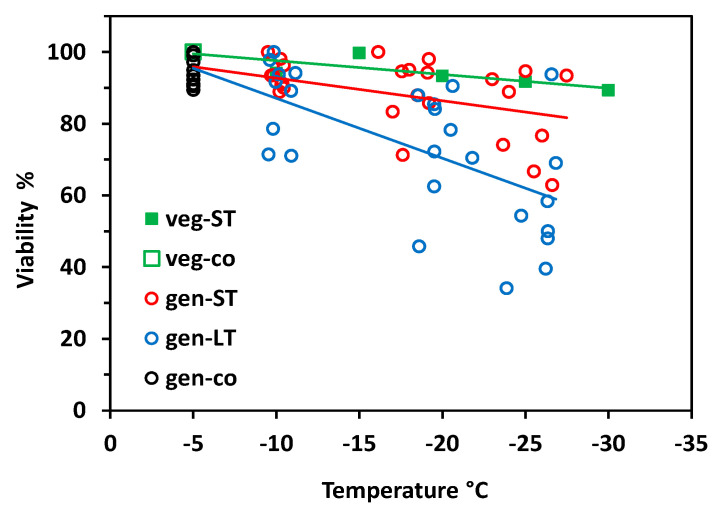
*Saxifraga moschata* Wulfen. Percent viability (TTC test) of control buds (co) and of vegetative (veg) and generative (gen) buds in the short-time (ST) and long-time (LT) experiment in winter. Vegetative buds (green squares): each symbol refers to the total of investigated vegetative buds pooled from 8–12 individuals per test temperature (ST experiment 1). Generative buds in control individuals (open black circles) and in individuals after ST (red circles) and LT (blue circles) exposure at different freezing temperatures in experiment 2; each symbol refers to a single individual. Trend lines indicate linear regressions.

Vegetative buds proved to be largely frost-hardy over the whole test temperature range in the short-time experiment (Figure 2). Viability still amounted to 90% after having been frost treated at −30 °C. Flower buds of control individuals—which were kept at −5 °C throughout—were viable between 89 and 100%. Flower bud viability significantly decreased with decreasing freezing temperatures in both experiments (short-time, ST: r^2^ = 0.28, *p* = 0.001; long-time, LT: r^2^ = 0.52, *p* < 0.001;), whereby LT freezing caused significantly more losses than ST freezing (ANCOVA, temperature*treatment, *p* = 0.003). In the ST experiment, flower bud viability was in the control range up to about −20 °C but fell to 70% in single individuals at lower temperatures. In the LT experiment, single individuals showed reduced flower bud viability already at −10 °C; below −20 °C, frost damage amounted up to 60%. Surprisingly, in a few individuals, a high proportion of flower buds survived the lowest treatment temperatures.

**Figure 3:**
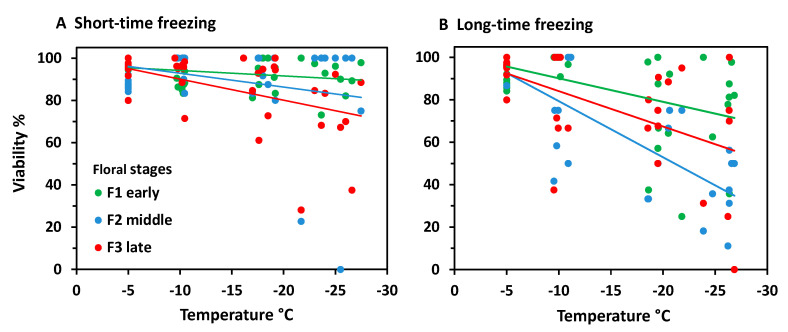
*Saxifraga moschata* Wulfen. Percent viability (TTC test) of floral winter buds in early (green), middle (blue) and late (red) developmental stages after (**A**) short-time and (**B**) long-time exposure at different freezing temperatures in experiment 2. For floral stages, see Figure 1. Each symbol refers to a single individual. Data at −5 °C are control values of the respective stage. Trend lines indicate linear regressions.

When analysing frost survival of floral developmental stages separately, early stages generally proved to be more freeze-resistant than advanced stages (Figure 3). In the ST experiment (Figure 3A) only early and late stages differed significantly (ANCOVA, temperature*stage, *p* = 0.013). Middle stages were in between and did not differ significantly either from early or late stages. Unlike in the LT experiment (Figure 3B), middle stages were significantly more susceptible than early stages (ANCOVA, temperature*stage, *p* = 0.006), whereas late stages were in between and did not differ significantly either from early or middle stages. Comparing the effect of frost treatments within the same stage, LT freezing caused a significantly higher loss of bud viability than ST freezing in all stages (ANCOVA, viability by treatment with temperature, *p* ≤ 0.04).

**Figure 4:**
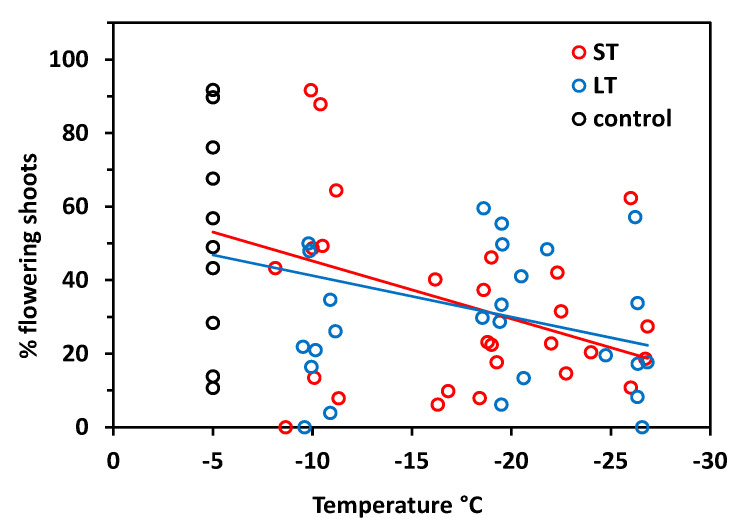
*Saxifraga moschata* Wulfen. Flowering frequency in control individuals (black circles), and in individuals after short-time (ST, red circles) and long-time (LT, blue circles) exposure in winter. Each symbol indicates the proportion of flowering shoots based on the total number of shoots (generative and vegetative) per individual. Trend lines indicate linear regressions.

In *S. moschata*, flowering frequency generally varies widely among individuals. The proportion of flowering shoots ranged between 11 and 92% in control plants and was accordingly variable in frost-treated individuals (Figure 4). Nonetheless, there was a significant relationship between freezing temperatures in winter and the flowering frequency in summer (ST: r^2^ = 0.20, *p* = 0.006; LT: r^2^ = 0.14, *p* = 0.026). Statistics did not reveal significant differences between ST and LT freezing (ANCOVA, temperature × treatment, *p* = 0.61), but there were aberrations in detail. In the ST experiment, the flowering frequency was still in the control range after exposure at about −10 °C and was reduced only below −15 °C. LT freezing caused reduced flowering frequency already from −10 °C downwards.

**Figure 5:**
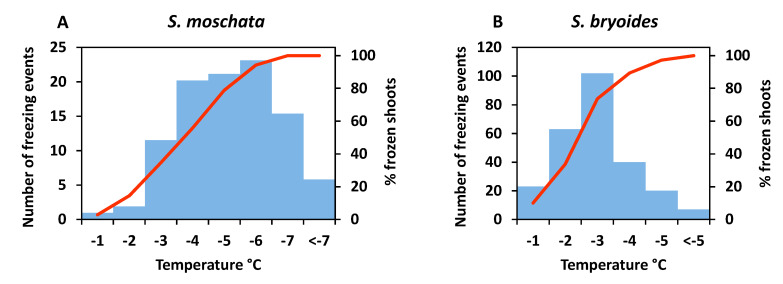
Number of freezing events (blue columns) and percent frozen shoots (red line) with successively decreasing freezing temperatures in an individual of (**A**) *Saxifraga moschata* Wulfen and (**B**) *Saxifraga bryoides* L., determined by IDTA.

Each single short-stem shoot in a *S. moschata* cushion froze separately. The freezing order appeared to be random, although at the beginning, more often, shoots at the cushion margin were nucleated. It was not before −1.7 °C that ice formation was detected in the first shoots (Figure 5A). The majority (77%) of shoots froze between −3 °C and −6 °C. The last freezing event was recorded at −8.9 °C. Under the experimental conditions, freezing of all shoots of the cushion took 2 h and 13 min. In shoots that had shown ice formation at freezing temperatures higher than −8.9 °C, no second freezing event could be detected in the course of the freezing treatment down to −40 °C, even if temperatures fell below the frost killing threshold of flower buds.

### 2.2. Effects of Winter Frost on Saxifraga bryoides

**Figure 6:**
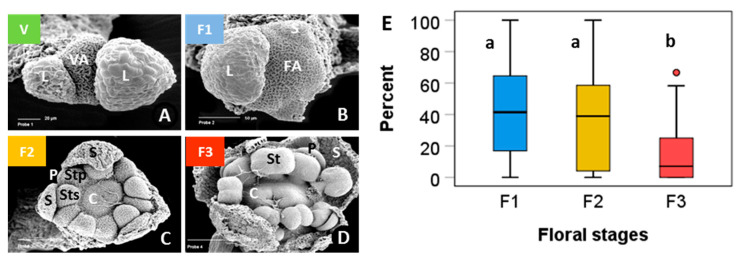
*Saxifraga bryoides* L. Bud stages in winter-dormant individuals at the time of the freezing experiments; green: vegetative (V); blue: early floral stages (F1); yellow: middle floral stages (F2); red: late floral stages (F3). (**A**) Vegetative shoot apex (VA) with leaf primordia (L) in alternate position; (**B**) floral apex (FA) shortly after the transition from vegetative to floral; the apical dome has flattened and sepal primordia (S) have emerged; (**C**) sepal and petal (P) primordia and two whorls of stamens in episepal (Sts) and epipetal (Stp) position have formed; carpel (C) margins appear as a crater-like structure. (**D**) Stamen (St) primordia have started to differentiate into filaments and anthers, carpels are forming a cone. Sepals that had begun to conceal the flower within were partly removed. (**E**) Percentage range of floral buds in different stages per individual; for details on the boxplot see Figure 1H; different lowercase letters indicate statistical differences among floral stages (*n* = 44 individuals; S1, S2 > S3, *p* ≤ 0.001, one-way ANOVA).

Buds of winter-dormant cushions were on average 88% ± 10 SD vegetative and 12% ± 10 SD floral. Development of floral buds ranged from early primordial to all floral organs basically preformed (Figure 6). The different developmental states were categorized into vegetative (Figure 6A), floral early (floral apex flattened and sepal primordia emerged, F1 in Figure 6B), floral middle (sepal and petal primordia and two whorls of stamens formed, F2 in Figure 6C), and floral late (all floral organs formed, F3 in Figure 6D). On average, 43% of the floral buds were in an early, 39% in a middle and 19% in a late floral stage (Figure 6E). As in *S. moschata*, the proportion of floral stages strongly differed among individuals. Most floral buds were either in stage F1 or F2, and significantly fewer buds were in stage F3 (F1, F2 > F3, *p* < 0.001, one-way ANOVA).

**Figure 7:**
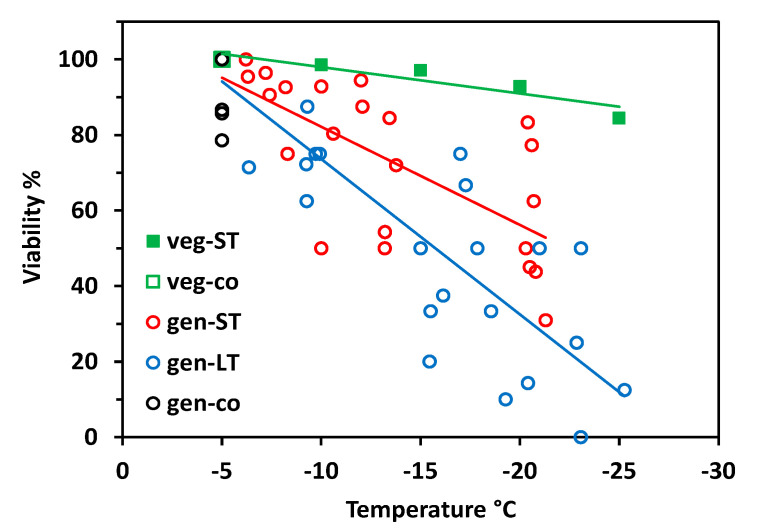
*Saxifraga bryoides* L. Percent viability (TTC test) of control buds (co) and of vegetative (veg) and generative (gen) buds in the short-time (ST) and long-time (LT) experiment in winter. Green squares: each symbol refers to the total of investigated vegetative buds pooled from 8–12 individuals per test temperature in ST experiment 1. Generative buds in control individuals (open black circles) and in individuals after short-time (red circles) and long-time (blue circles) exposure at different freezing temperatures in experiment 2; each symbol refers to a single individual. Trend lines indicate linear regressions.

Vegetative buds were hardly affected by ST freezing. Bud viability was 100% in control individuals, dropped to 84% at −25 °C (Figure 7), and to 80% at −35 °C (data not shown). Generative buds of control individuals that were kept at −5 °C throughout were viable between 79 and 100%. Floral bud viability significantly decreased with freezing temperatures, both in the ST experiment (r^2^ = 0.58, *p* < 0.001) and the LT experiment (r^2^ = 0.77, *p* < 0.001). As in *S. moschata*, LT freezing caused significantly higher damage than ST freezing (ANCOVA, temperature*treatment, *p* < 0.001). Remarkably, flower bud viability dropped below the control range already around −10 °C—in few individuals in the ST experiment but in most individuals in the LT experiment. At −20 °C and below, LT freezing killed 50 to 100% of flower buds per individual.

**Figure 8:**
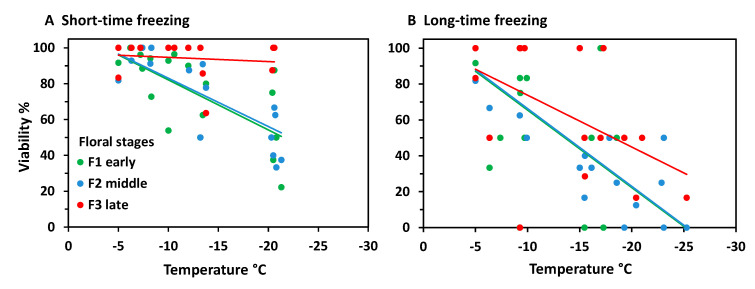
*Saxifraga bryoides* L. Percent viability (TTC test) of floral winter buds in early (green), middle (blue) and late (red) developmental stages after (**A**) short-time and (**B**) long-time exposure at different freezing temperatures in experiment 2. For floral stages see Figure 6. Each symbol refers to a single individual. Data at −5 °C are control values of the respective stage. Trend lines indicate linear regressions.

At the stage level, the most advanced flower buds appeared to be more freezing tolerant than the less developed ones (Figure 8). Differences were most pronounced in the ST experiment (Figure 8A); (ANCOVA, temperature × stage, *p* ≤ 0.001), but—due to a wide dispersion of individual values—not significant in the LT experiment (Figure 8B). After ST freezing at temperatures slightly below –20°C, viability of F3 buds was still in the control range, whereas F2 and F3 buds were damaged up to 80%. Long-time freezing markedly damaged F3 buds from −15 °C and downwards; F1 and F2 buds were already damaged below −5 °C. At −25 °C, the survival rate was still about 20% in F3 buds, but zero in F1 and F2 buds. Comparing the effect of frost treatments within the same stage, long-time freezing caused a significantly higher loss of bud viability than ST freezing in all stages (ANCOVA, viability by treatment with temperature, *p* < 0.007).

**Figure 9:**
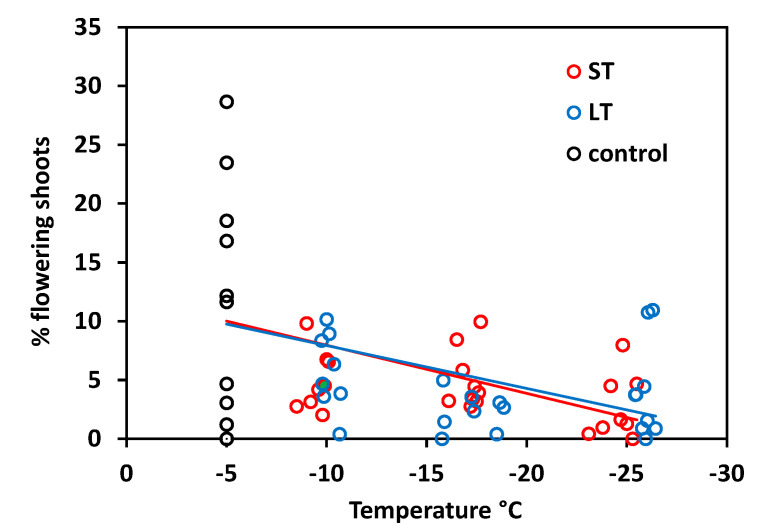
*Saxifraga bryoides* L. Flowering frequency of control individuals (black circles), and individuals after short-time (ST, red circles) and long-time (LT, blue circles) exposure that had been exposed to different freezing temperatures in winter. Each symbol indicates the proportion of flowering shoots based on the total number of shoots (generative and vegetative) per individual. Trend lines indicate linear regressions.

The flowering frequency varied between 0 and 29% in control plants, but attained only 11% at the most in individuals that had been exposed to freezing temperatures in winter (Figure 9). On the basis of control values, the flowering frequency significantly decreased with temperature both after ST freezing (r^2^ = 0.22, *p* = 0.004) and LT freezing (r^2^ = 0.19, *p* = 0.008) without significant differences between treatments (temperature × treatment *p* = 0.81, ANCOVA). Excluding the control values in the regression analysis, there was no significant relationship between the freezing temperature and flowering frequency in both treatments (ST r^2^ = 0.10, *p* = 0.12; LT r^2^ = 0.03, *p* = 0.40). This means that the flowering frequency was reduced to a similar degree over the whole temperature range from −10 to −25 °C.

The cushion of *S. bryoides* froze in a characteristic pattern in which for each single short-stem shoot a separate freezing event could be spotted. In each shoot, ice initially formed in a single or several leaves; thereafter, all leaves froze successively within a short time. The order of shoots freezing in the cushion was not so clear but it seemed that more exposed or marginal shoots froze earlier. Already at −0.5 °C, ice formation was detected in the first shoots (Figure 5B). At −2.3 °C, 50% of all shoots were frozen and freezing peaked between −2 and −3 °C, where ice formed in 44% of the shoots. The last freezing event was recorded at −6.7 °C. Under the experimental conditions, freezing of all shoots of the cushion took 1 h and 22 min. At lower freezing temperatures or at freezing temperatures that were lethal for flower buds, no further freezing event could be registered.

### 2.3. Effects of Winter Frost on Ranunculus glacialis

**Figure 10:**
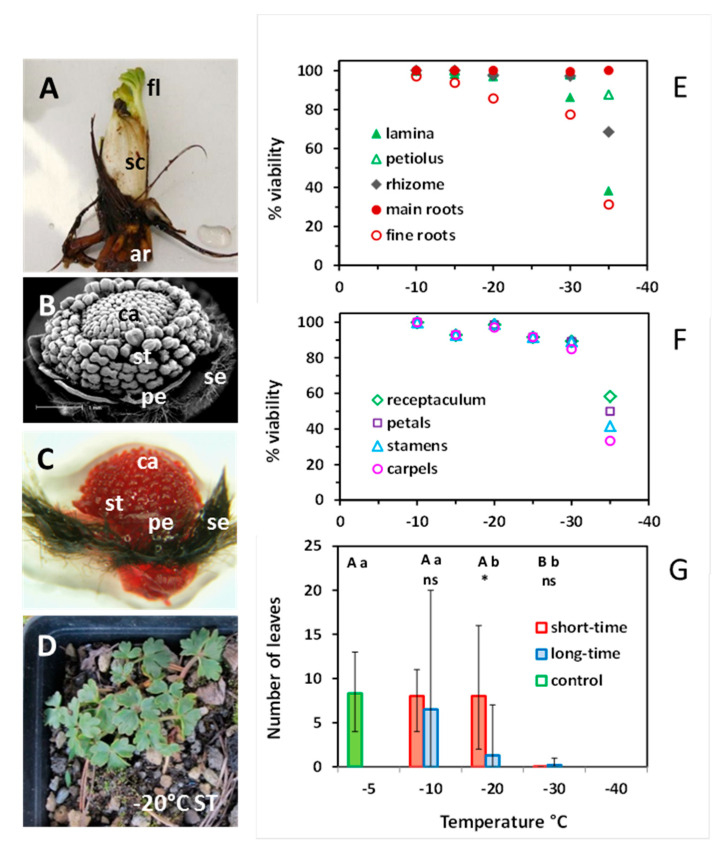
*Ranunculus glacialis* L. (**A**) Belowground winter bud with scale-like leaves (sc) enclosing foliage leaves (fl) and a determinate inflorescence (not visible from outside); foliage leaves started to sprout and turned green during the latency period between freezing treatment and damage assessment; ar adventitious roots arise from the short rhizome. (**B**) Developmental state of the preformed terminal flower in winter; pe petal, se sepal, st stamens, ca carpels. (**C**) flower bud after vital staining with TTC; sepals are covered with dark brown trichomes, which did not stain. (**D**) Regrowth of an individual after short-time (ST) exposure to −20 °C in winter (experiment 2). Percent viability (TTC test) of (**E**) vegetative organs and (**F**) floral organs after ST exposure to different freezing temperatures in winter (experiment 1). (**G**) Mean number of leaves per individual sprouting in spring in control plants kept at −5 °C (green bar), and after ST (red bars) and LT (blue bars) exposure at −10, −20 and −30 °C in winter (experiment 2). Error bars: max and min. Statistical differences (*p* ≤ 0.05) among temperatures are indicated by upper case letters for the short-time experiment, and by lower case letters for the LT experiment (one-way ANOVA). Statistical differences between the ST and LT treatment at the same temperature were determined by *t* test: * *p* = 0.04, ns not significant.

The overwintering below-ground ramets consist of a short rhizome with adventitious roots and a terminal bud wrapped by scale-like leaves (Figure 10A). The scales surround the foliage leaves and a determinate inflorescence with a variable number of flower buds. The scales do not appear aboveground and senesce around anthesis in summer. Flower buds develop during the previous growing season and pass winter in an advanced state (Figure 10B,C): sepals are covered with dark-brown villous hairs and fully envelope the inner flower organs; petals are still underdeveloped; anther filaments and pollen sacs start to differentiate; each carpel contains a single pre-meiotic ovule (not visible in the picture) and the carpel tips (the future stigmata) begin to elongate.

In the short-time experiment 1, the main roots were the most freeze-resistant vegetative organs; they survived −35 °C without damage (Figure 10E). The rhizome remained undamaged down to −30 °C; at −35 °C, viability dropped to 68%. The lamina of foliage leaves was the most vulnerable vegetative structure: 14% were damaged at −30 °C, and 62% at −35 °C. Viability of floral organs slightly decreased from 100% at −10 °C to 90% at −30 °C (Figure 10F). Distinct frost damage only occurred at −35 °C, whereby carpels proved to be the most vulnerable structures.

The individuals available for the regrowth experiment 2 were of small size and unfortunately did not flower—control plants included. Nevertheless, the number of foliage leaves per individual sprouting in summer provides information about the ability to survive winter frosts (Figure 10D,G). After ST freezing in winter, regrowth in summer did not differ from control plants down to −20 °C, but failed after −30 °C. After LT freezing, regrowth was in the control range at −10 °C, significantly reduced at −20 °C and nearly zero at −30 °C.

## 3. Discussion

Vegetative buds of *S. moschata* and *S. bryoides* survived even the lowest test temperatures of −30 and −35 °C largely undamaged, which confirms the generally high winter freezing resistance of vegetative organs in high-mountain plants [29,31,33,57]. Conversely, the flower buds were not sufficiently frost hardy; their viability decreased significantly with increasing frost intensity and duration. The flower buds of the nival species *S. bryoides* turned out to be particularly susceptible to freezing. Even flower buds of control plants which had been kept uncovered at −5 °C throughout lost on average 5% of their viability. Moderate freezing temperatures around –10 °C killed on average 20% (short-time freezing) and 26% (long-time freezing) of the flower buds per individual; below −20 °C, the mean losses were 47 and 75%, respectively. This means that individuals without secure snow cover would lose a high percentage of preformed flower buds in winter. Remarkably, the same discrepancy in freezing resistance between vegetative and generative buds in winter was also found in summer [19]; in that study, *S. bryoides* was among the most freezing resistant high-mountain species regarding the vegetative shoots (LT_10_ at −10.6 °C, LT_50_ about −12 °C), whereas generative shoots suffered damage from –1.5 °C downwards (LT_50_ around −4 °C) at bolting and anthesis. Winter-dormant flower buds of *S. moschata* have proved to be less vulnerable—at least in the short-time experiment: percent mean damage was largely in the control range down to −20 °C, and amounted to 19% around −25 °C. During long-time freezing, however, flower bud losses dropped below the control values already at −10 °C, and reached on average 38% around −25 °C.

Though it is a well-known fact that generative structures are more frost susceptible than vegetative ones also during winter dormancy [31], the relatively low freezing resistance of generative buds in both saxifrages was surprising. It might have been expected that cushion plants, whose shoots overwinter aboveground, and in the case of *S. bryoides* and *S. moschata* may be snow-free at exposed sites, either avoid tissue freezing by supercooling or tolerate extracellular ice formation and freeze-dehydration. In both saxifrages, there is only little capacity for supercooling of whole individuals in winter. Infrared thermography showed that ice formation in the shoots peaked between −2 and −3 °C in *S. bryoides*, and between −3 and −6 °C in *S. moschata* without further freezing events below −7 and −9 °C, respectively. Freezing occurred in about the same range as observed during the growing season [19]. As in summer, shoots froze autonomously [40]. In summer, the spread of ice from one shoot to the next might have been interrupted by thermal insulation inside the cushion where the individual shoots arise. Under the experimental conditions in winter, a thawed soil was simulated. Freezing pattern of the cushion will change when cushions freeze again after surficial daytime thawing in the sun. As observed in daytime-thawed apple trees [58], ice will then always spread from the still-frozen belowground organs once the shoot temperature drops below 0 °C. Upon ice nucleation, mostly in the outer leaves of an individual shoot, ice spread in all parts of the shoot within seconds. In winter, it was not possible to differentiate between vegetative and generative shoots from the outside, nor we could observe where exactly ice accumulated. In shoots and mature leaves, extracellular tissue freezing might have taken place. Primordial tissues at the shoot tip, however, lack intercellular space that could accommodate ice. Since lethal intracellular freezing can be excluded at these high freezing temperatures, ice formation outside of the primordial structures is assumed. This extra-organ freezing [59] might have led to freeze dehydration of primordial tissues. We were unable to register any freezing processes in the lethal temperature range of buds. Either the energy released was below the resolution of our infrared camera or the primordial tissues do not freeze intracellularly and frost damage would supposedly be caused by increasing duration and intensity of freeze dehydration. Nevertheless, in both *Saxifraga* species, generative structures obviously are much more susceptible to the processes triggering frost damage than vegetative structures. We had expected that flower buds in an advanced stage of differentiation would be more susceptible to frost than early primordial buds, which lack specific structures and resemble more the leaf primordia in a vegetative bud. These expectations were confirmed only for *S. moschata* in the short-time experiment (early < middle < late) but not in the long-time experiment. In *S. bryoides*, both in the long-time and short-time experiment early and middle stages were more susceptible than advanced stages. We do not have a conclusive explanation for this result. It is conceivable that in advanced stages of flower development with already more developed cell walls, cellular freeze dehydration is better tolerated.

The decrease of flower bud viability with increasing freezing strain in winter was not reflected by the flowering frequency in summer to the same extent. There was a significant decrease of flowers per individual with increasing freezing temperatures, but there was no significant effect of the exposure time. The main reason is that the number of floral shoots varies considerably among individuals of both *Saxifraga* species, as evidenced by the control values in the present study and by earlier studies [14,60]. The strong individual variation in the number of flowers clearly overlayed the frost effect. Additionally, it is conceivable that new vegetative shoot apices shift to floral at the beginning of the growing season when too many flower buds have been killed during winter. This could particularly apply to *S. bryoides*, whose flower buds develop largely or even completely in the year of anthesis [54]. Within an individual cushion, up to three cohorts of flowers appear at staggered intervals. The first cohort starts with basically differentiated flower organs (as found in the winter samples here), whereas cohort 2 and 3 start from the beginning. Newly formed flower buds might compensate for some of the losses of flower buds in winter and thus preserve the opportunity to generate offspring. However, due to an about 8 weeks prefloration period and another 7 weeks for seed maturation [14,52], a restart would only mature seeds at early thawing sites where the growing season is long enough. Despite the vagueness regarding the number of generative buds coming into flower, the main differences in freezing resistance between *S. moschata* and *S. bryoides* became evident also in the flowering frequency. In *S. bryoides*, even moderate frost in winter negatively impacted flowering in summer, whereas in *S. moschata*, the aftereffects of winter frost were less pronounced.

In *R. glacialis*, all plant organs are more or less equally exposed to winter frost. The cryptophyte overwinters as rhizome bud about 5 to 10 cm below ground, roots are growing deeper. The freezing resistance of the rhizome and the renewal buds can be considered most decisive for the survival of an individual, whereas stem borne roots may regenerate. In the short-time experiment, fine roots and leaves were the most freezing-sensitive organs, whereas main roots, rhizomes and flower buds remained largely undamaged down to −30 °C. Nonetheless, regrowth in summer was almost nil after exposure to such low temperatures but was still in the control range at −20 °C. Permanent frost is hardly tolerated by this species, as shown in the regrowth experiment. One week at −20 °C killed most of the individuals. This confirms that *R. glacialis*—though occurring at the elevational and latitudinal limits of higher plant life—is not adapted to frost exposed sites but requires a continuous winter snow cover. Unprotected plants would hardly survive even normal mountain winters in which air temperatures can regularly drop below −20 °C. The moderate freezing resistance in winter stands in contrast to the high freezing resistance in summer [16,19,20,22]. In the nival zone, plants regularly experience subzero temperatures during clear summer nights. In the case of ice nucleation—which starts at mean at −2.6 °C—extracellular freezing leads to the accumulation of huge ice masses in the intercellular spaces of the spongy tissue of the leaves, accompanied by cytorrhysis and freeze dehydration [16]. Following moderate night frost, plants regain their full photosynthetic performance immediately after thawing in the morning. Thus, *R. glacialis* tolerates regular freeze–thaw cycles during the metabolically active state at full turgidity. This is similar to the habitat conditions of high-elevation plants in tropical, subtropical and arid mountains, where, during active growth, night time air temperatures regularly fall to −4 to −8 °C or even lower and plants have evolved different strategies to cope with freezing [31,61,62,63,64,65,66]. Unfortunately, we do not have data about ice nucleation in winter; however, freezing tests in summer have shown that there is no anatomical ice barrier in *R. glacialis*; ice spreads unhindered from the root into all parts of the shoot at subzero temperatures [67]. On the assumption that the supercooling capacity is low and tissues stay frozen for a longer time period, they might suffer frost damage due to long-lasting freeze-dehydration. Further investigations are necessary to prove this hypothesis.

In regions with cold winters, the risk of frost injury strongly depends on whether or not plants are protected by a permanent snow cover of an adequate depth [1,31,41,68,69]. The lower the ambient air temperatures, and the longer the freezing periods, the thicker the snow cover must be to exert an insulating effect [41]. At snow-free sites, winter frost penetrates into the ground. In the upper soil layers, the main growing zone of prostrate plants, freezing temperatures largely follow the fluctuation of the ambient air temperatures [28,70]. In recent years, there has been a trend towards a reduction of winter snow cover duration in temperate and arctic regions [29,69,71,72,73,74,75]. In the Austrian Alps, substantial snow falls usually do not occur before January [17]. However, during polar cold waves in the months before, free air temperatures in the alpine and nival zone may drop below −20 °C and keep the soil frozen for weeks [25,28,76,77]. The same may happen in late winter when cold waves follow a warmer period, during which shallow snowpacks at wind-blown sites become thinner or melt away. As a consequence, unprotected plants are directly exposed to severe freezing temperatures for some time. As reported by many studies, and shown in the present study, vegetative parts of high-alpine and nival cushion plants are sufficiently frost hardy to survive such severe frosts and thus ensure the persistence of single individuals in their habitat. However, they are at risk to lose flower buds and thus the possibility to produce progeny in the year to come. Thus, the reproductive success of high-mountain plants is not only endangered by episodic spring and summer frosts but also by winter frosts due to insufficient snow coverage. Changes in the snow cover regime in winter have already been shown to impact the flowering frequency in summer [13,75,78,79]. Since the response is species-specific, range shifts of single species leading to changes in the composition of plant communities are to be expected [28]. In the case of the *Saxifraga* species studied here, the picture is split. The cushions of *S. bryoides* and *S. moschata* are sufficiently frost hardy to resist severe winter frosts, which provides an advantage over species, which are unable to colonize exposed and frost dominated sites—such as the chionophyte *R. glacialis*. However, the persistence of the species in a habitat will also depend on whether sufficient individuals occur in snow-protected sites to ensure flowering and recruitment by seeds.

## 4. Materials and Methods

### 4.1. Plant Material and Collection

**Figure 11:**
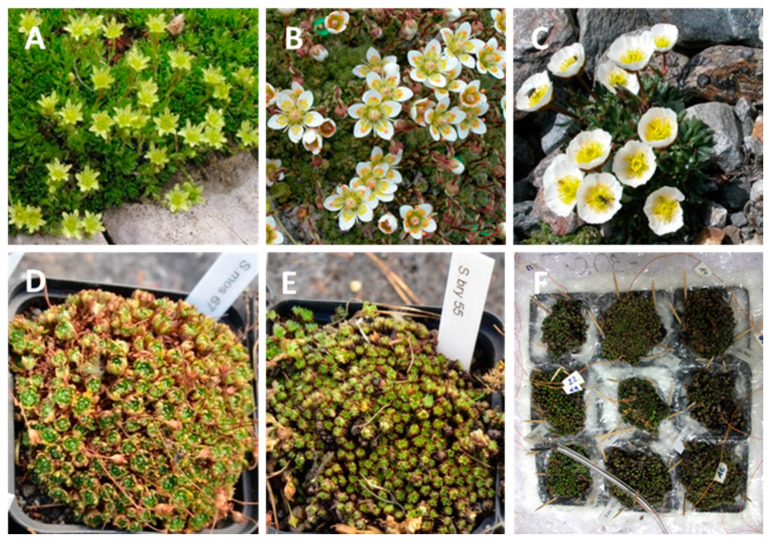
(**A**) *Saxifraga moschata* Wulfen, (**B**) *Saxifraga bryoides* L. and (**C**) *Ranunculus glacialis* L. at full bloom in summer. (**D**) *S. moschata* and (**E**) *S. bryoides* in the dormant state during winter. Cushions consist of densely arranged short-stem shoots with naked terminal buds, formed by inwardly bent foliage leaves. It is not visible from outside whether a bud is vegetative or floral. (**F**) freezing unit in experiment 2 consisting of nine potted *S. bryoides* individuals placed in a styrofoam box; the space between the pots is filled with foam material, and the rims of the pots are isolated with an air bubble film. Thin, brown cables are thermocouples recording temperatures within the buds; the thicker cable records the ambient temperature within the chest freezer.

The study was carried out on three high-mountain species that are common in their respective habitats and grow at sites with different snow depths in winter. *Saxifraga moschata* Wulfen (Figure 11A) is a perennial semi-evergreen cushion plant which occurs frequently in most European mountain systems, the Caucasus and the Altai mountains from the alpine to the nival zone (1500 m to 4200 m a.s.l., [80]). The pioneer species prefers base-rich substrates and grows on stable and unstable scree and in rocky swards [80] with more or less snow protection in winter [60]. The compact hemispherical cushions consist of densely foliated orthotropic short-stem shoots of the columella type [81]. In late August, terminal apices of a part of the vegetative short-stem shoots become floral and initiate inflorescences with one to five flower buds each. Flower buds enter winter dormancy in different developmental stages from early primordial to all floral organs basically differentiated [82]. Inwardly bent foliage leaves that have developed during the preceding growing season form naked winter buds (Figure 11D) which enclose either a vegetative shoot apex or floral structures.

*Saxifraga bryoides* L. (Figure 11B) is a perennial semi-evergreen cushion plant typical of the plant assemblages from the subnival (i.e., the alpine-nival ecotone) to the nival zone throughout the central European Alps [83]. The species prefers siliceous substrates and grows mainly in open habitats on scree and solid rock with little snow cover in winter [84]. *S. bryoides* forms semi-compact cushions with densely foliated, orthotropic short-stem shoots of the columella type in the centre and creeping shoots with lateral rosettes-shoots in the periphery [81]. From August onwards, apices of a low proportion of vegetative shoots become floral and develop into terminal flower buds. Most flower buds enter winter-dormancy in an early development stage [54]. As in *S. moschata*, naked winter buds are formed which are either vegetative or floral (Figure 11E). *Ranunculus glacialis* L. (Figure 11C) is an arctic-alpine perennial herbaceous cryptophyte, widely distributed in European and Scandinavian mountains at higher elevations, and in the Arctic at sea level [85,86]. In the Central European Alps, the species occurs from the subnival to the nival zone at sparsely vegetated sites with sufficient snow cover in winter [84]. The overwintering belowground rhizome system consists of individually rooted bud-like ramets (see Figure 10A) with alternate arranged leaves and a uni- to multiflorous inflorescence [55,87,88]. Winter-dormant terminal flower buds are in an advanced development state [55].

Individuals of *S. moschata* were sampled on Mt. Hafelekar in the calcareous mountain range north of Innsbruck (2350 m a.s.l., 47°18′ N, 11°23′ E). Individuals of *S. bryoides* and *R. glacialis* were collected in the subnival glacier foreland of the Stubai Glacier (2880 m a.s.l., 46°59′ N, 11°07′ E). Sampling of the widely scattered individuals is not possible in high winter at higher elevations. Either the plants are buried below a huge frozen snowpack or—when snow cover is shallow—soil and root-balls are frozen hard. Therefore, whole plant individuals were excavated with root bales and potted into plastic containers (8 × 8 cm) in an alpine soil mixture supplemented with soil from the natural site before the onset of winter conditions in late October. Immediately after potting, plants were transferred to the Alpine Research Garden of the University of Innsbruck at 1950 m a.s.l. on Mt. Patscherkofel (experiment 1), or to the mountain station of the Services for Torrent and Avalanche Control Tirol in Haggen at 1600 m a.s.l. (experiment 2). In January, a few days before the start of the freezing experiments, pots were dug out from below the snow and transferred to the lab. To avoid frost dehardening plants were kept in a freezer at −5 °C until the onset of freezing treatments. This temperature was chosen as plants usually experience temperatures between zero and −5 °C when they pass winter below the snow [25,68,70].

### 4.2. Freezing Experiments

Exposure to freezing temperatures took place in temperature-controlled chest freezers (Liebherr, Lienz, Austria). Temperature inside the freezing compartment was controlled using adequately programmed software (LabView 2012, NI, Austin, TX, USA) that realizes user-defined cooling profiles with a temperature accuracy of ±0.2 Kelvin (K) (for details, see [15]). Temperature control was put into effect by targeted heating against the permanently cooling chest freezer by use of PTC heaters (Nimbus B200, DBK Austria, Krems, Austria). Temperature equalization within the freezing compartment was achieved by ventilators. The ambient temperature inside the freezing chamber was monitored with a thermocouple (Type T, wire diameter 0.32 mm).

Freezing experiments were conducted in two different winters, referred to as experiment 1 (winter 2011/12) and experiment 2 (winter 2015/2016). The plant material was subjected to step cooling procedure consisting of a cooling down phase, a cooling phase at the target temperature and a warming-up phase. Target freezing temperatures were set in 5 K (experiment 1) and 10 K (experiment 2) steps across the temperature range from −10 to −30(35) °C. In a single freezing experiment, cooling started at −5 °C and temperatures were lowered at a rate of −3 K·h^−1^ down to the target temperature, which is recommended for temperature-controlled freezing experiments [39]. Plants remained either for one night (5 h, short-time experiment, ST, simulating a single frost night) or for one week (168 h, long-time experiment, LT, simulating a frost period of one week) at the target temperature. During the subsequent warming-up phase, temperatures rose at a rate of 3 K·h^−1^ until −5 °C was reached again. Plant temperatures were continuously recorded using fine-wire copper-constantan thermocouples (welding spot diameter 0.15 mm). The welding spots were inserted within the buds (saxifrages) or placed beside the *Ranunculus* buds. For each freezing series, three to eight thermocouples per test set were mounted (Figure 11F). Additional thermocouples were placed in the root zone. Temperatures are presented in °C and temperature differences in Kelvin (K) as is the custom in bioclimatology [89].

In experiment 1, only short-time treatments in 5K-steps between −10 and −35 °C took place to test for direct frost effects. Per test temperature, 8–12 potted individuals of each *Saxifraga* species and four to six ramets of different *R. glacialis* individuals were placed in flat plastic tubs and transferred to the respective chest freezer. For *R. glacialis*, ramets were excavated, placed on moist filter paper and wrapped in aluminium foil. After freezing, the ramets were repotted to monitor regrowth. Ten individuals per species remained at −5 °C and later served as controls.

In experiment 2, short-time and long-time freezing treatments were performed at –10, −20, and −30 °C, to determine both the direct frost effect on flower buds in winter, and the impact on the flowering frequency in summer (*n* = 9 individuals per species, test temperature and test objective). To avoid frost effects on the roots of the saxifrages, which might have influenced shoot development and flowering later on, only the aboveground tissues were exposed to the freezing temperatures. Nine pots per species and temperature run were placed in thermally insulated boxes from styrofoam (Figure 11F); the space between the pots was filled with foam material, and the rim of the pots were isolated with an air bubble film. Small heating mats (ThermoLux 6W, Witte+Sutor GmbH, Murrhardt, Germany) on the bottom of the boxes, which were controlled by a temperature sensor (Universal Thermostat UT300, eQ-3 AG, Leer, Germany) kept the temperatures in the root space by an average of 3 K (−10 °C), 10 K (−20 °C) and 15 K (−30 °C) above the target temperature, and thus outside the damage range for roots of alpine plants [33]. The disadvantage of a warmer root space was that, due to a temperature gradient between soil and aboveground tissues, individual plant temperatures deviated from the target temperature. Therefore, in the results, data on frost damage and flowering frequency are given separately for each individual and refer to the mean minimum temperature experienced by the aboveground organs during the temperature treatment. For the cryptophyte *R. glacialis*, it was not possible to protect the roots from possible frost effects. In this case, the potted individuals were exposed to freezing temperatures without thermal isolation. Control individuals (*S. bryoides,* two sets of *n* = 10 individuals each, one for the TTC-test and one for the assessment of the flowering frequency; *S. moschata,* two sets of *n* = 9 individuals each; *R. glacialis* one set *n* = 9 for the regrowth test) remained at −5 °C in the meantime.

### 4.3. Assessment of Viability and Regrowth Test

Immediately after the freezing treatment, those individuals that were examined for direct frost effects were, together with control individuals, transferred to growth chambers in experiment 1 (temperature day/night 10/6 °C, PGC-GL, Percival Scientific Inc., Perry, IA, USA), or to a temperature controlled green house in experiment 2 (day/night 15–20/10 °C) for two weeks. This long time lapse between freezing treatment and damage analysis is necessary to develop damage symptoms in the winter dormant state [90]. Individuals whose flowering frequency was determined in summer were transferred to the mountain station of the Services for Torrent and Avalanche Control Tirol in Haggen at 1600 m a.s.l. and placed in perforated boxes below the snow. The plant boxes were permanently video monitored (BK-Elektronik, Natters, Österreich). This way, despite remoteness of the site, periods with snow cover and the onset of flowering could be exactly ascertained.

#### 4.3.1. TTC Staining

Direct frost injuries were determined by vital staining using a 0.5% TTC solution (2,3,5-Triphenyltetrazoliumchloride, Merck, Darmstadt, Germany), pH 7–8 following earlier protocols [22,90]. Samples to be examined were placed in 10 mL glass test tubes filled with the TTC solution, and infiltrated under vacuum (vacuum pump LKC251E, Saskia Hochvakuum+Labortechnik GmbH, Illmenau, Germany) in an exsiccator. Subsequently, the test tubes were filled up to the rim with TTC solution and tightly closed. The samples were incubated for 24 h at room temperature in darkness. During the incubation, living tissues and cells turned red as a result of the activity of dehydrogenases that transformed colorless TTC into the red triphenylformazan. After staining, the samples were washed with distilled water, conveyed into glycerol (86%, ROTIPURAN p.a., Roth, Karlsruhe, Germany) and stored in the dark at room temperature until examined for frost damage. Buds were rinsed with distilled water, dissected under a stereo microscope and the respective structures were scored as living (fully or largely colored red) or dead (scarcely colored or undyed).

In experiment 1, the freezing resistance of vegetative shoots of the saxifrages and of all belowground organs of *R. glacialis* was assessed. For each *Saxifraga* species, a large number of buds was randomly excised from 8 to 12 individuals per test temperature and pooled together for the TTC-test. Out of each sample pool about 30 (*S. moschata*) and 40–70 (*S. bryoides*) vegetative buds were analysed. The shoot apex with leaf primordia, the five innermost mature leaves and the stem were checked for viability, and the percentage of frost damage was calculated. For *R. glacialis*, percent viability of the terminal flower (separately for receptacle, sepals, petals, stamens, carpels), foliage leaves (separately for lamina and petioles), rhizome and roots of four to six individuals per test temperature was evaluated.

In experiment 2, the freezing resistance of flower buds in the saxifrages was examined. Per test temperature and exposure time *n* ≥ 120 (*S. bryoides*) and *n* ≥ 80 (*S. moschata*) buds were randomly excised from each individual (*n* = 9) and stained with TTC. In winter, vegetative and generative buds are indistinguishable from outside (see Figure 11D,E). Furthermore, the percentage of generative buds strongly varies among individuals. Thus, a considerable number of buds had to be sampled and dissected to gain a sufficient number of flower buds. The yield of flower buds per individual sample ranged between 16 and 40 for *S. moschata* and between 2 and 35 for *S. bryoides*. The percentage of vital flower buds was expressed on the basis of all investigated flower buds per individual.

#### 4.3.2. Assessment of Flowering Frequency and Regrowth

At the time when the *S. moschata* and *S. bryoides* plants were in full bloom, all inflorescences per individual were harvested and counted. A digital photo of each individual was then taken and the total number of shoots was determined using an image analysis programme (ImageJ, U.S. National Institutes of Health). The flowering frequency was calculated as a percent of the total number of shoots per individual. For *R. glacialis*, the flowering frequency was not ascertainable because the individuals did not flower—this was also the case for the control plants. Obviously, most of the excavated ramets were too young and still in the vegetative phase. Unfortunately, it was not visible from outside whether a ramet is vegetative or floral. Thus, only the regrowth capacity was determined by counting the number of basal leaves that appeared above-ground in summer.

### 4.4. Infrared Differential Thermal Analysis (IDTA)

Freezing events were monitored in individual cushions of the saxifrages with a digital infrared camera (T650SC, FLIR Systems, Danderyd, Sweden; lens: 5.8 × 100 µm, frame rate: 15 Hz). This infrared camera has a thermal resolution of 0.2 mK. Freezing treatment and processing of the IR images with the software FLIR ResearchIR Max (version 4.20.2.74, FLIR Systems) was conducted as described earlier using infrared differential thermal analysis (IDTA) [38,91]. The freezing treatment took place in a temperature-controlled chest freezer as described above. Freezing was started at an ambient temperature of +2 °C. After thermal equilibrium, cushions were cooled at a mean rate of –6 K.h^−1^ down to a target temperature (*S. bryoides* −30 °C and *S. moschata* −40 °C, respectively) which was below the frost killing temperature. As soon as the cushion temperature dropped below 0 °C IR video clips were recorded throughout.

### 4.5. Statistics

Relationships between the freezing temperature, percent bud viability and flowering frequency were determined by linear regression analyses. For testing statistical differences between treatments (ST, LT freezing) and among floral stages within the same treatment, regression slopes were compared by analysis of covariance (ANCOVA) with the treatment or stage as fixed factors (independent variables) and temperature as a cofactor. This statistical method tests for the equality of regression coefficients. Statistical difference among regression slopes is given when the independent variable significantly interacts with the covariate [92,93]. Differences among the relative frequency of floral stages per individual were tested by one-way ANOVA followed by Bonferroni post hoc test. All tests were made with the statistical package SPSS (IBM, New York, NY, USA) at the critical level of significance α = 0.05.

## 5. Conclusions

On the example of three plant species (the cushion plants *Saxifraga moschata* Wulfen, *Saxifraga bryoides* L. and the cryptophyte *Ranunculus glacialis* L.), colonizing higher elevations in the European Alps, we could show that severe winter frosts impact flower bud survival and consequently flowering frequency and seed output in the season to come. Vegetative organs of the saxifrages turned out to be sufficiently frost hardy to largely survive even the lowest temperatures occurring at the natural sites. Floral buds, however, suffered frost damage already at moderate freezing temperatures, whereby they were affected to different extents—depending on the species, the developmental stage and frost severity and duration. Since winter buds did not supercool, the damages were very likely caused by freeze dehydration. In the cryptophyte *R. glacialis*, vegetative and reproductive structures were similarly frost resistant and did not survive extreme frosts. These findings demonstrate that high-mountain plants are not uniformly frost hardy in accordance with the prevailing climatic extremes. The life form (chamaephyte versus cryptophyte) but also evolutionary adaptation of a single species to the (micro-)habitat conditions play an essential role in the potential for frost damage. The diverse topography of high mountains offers a mosaic of climatic niches where species with similar requirements co-occur. Winter frosts hardiness is among the essential features determining whether or not a species needs snow-sure sites in winter to successfully grow and produce offspring in summer.

In the predicted scenario of climate warming, a high turnover rate in mountain environments is expected [94]. Models predict that plant diversity will initially increase with the retreat of glaciers because of the upward migration of lower-elevation species. Glacier extinction, however, will be followed by a decrease in species richness due to range losses of species inhabiting proglacial environments [95]. However, provided that mountains are high enough and drought effects do not play a role, the geo-diversity of alpine terrain reduces the risk of habitat loss [1]. Current simulations should therefore also consider snow cover dynamics and temperatures at ground level throughout the year—which are needed to develop realistic scenarios on the spatio-temporal dynamics of high-mountain communities in response to climate change [1,28]. Changes in species composition will not only depend on climate warming effects but also on the survival of generative structures as a basis for reproductive fitness under a changed annual snow and frost regime in both, upslope moving species and local species.

## Data Availability

Not applicable.
